# *SpatDistCalib*: a GUI Python software for spatial-distortion correction of 2D detectors using splines

**DOI:** 10.1107/S160057672300225X

**Published:** 2023-04-25

**Authors:** William Chèvremont

**Affiliations:** a ESRF – The European Synchrotron Radiation Facility, 71 Avenue des Martyrs, 38043 Grenoble, France; Lund University, Sweden; Keele University, United Kingdom

**Keywords:** 2D detector systems, spatial-distortion correction, regular calibration grids, graphical user interface Python software, splines

## Abstract

CCD-based X-ray detector systems often suffer from spatial distortions. Reproducible distortions can be quantitatively measured with a calibration grid and described as a displacement matrix or as spline functions. The measured distortion can be used afterwards to undistort raw images or to refine the actual position of each pixel, *e.g.* for azimuthal integration. This article describes a method using a regular grid, not necessarily orthogonal, to measure the distortions. The graphical user interface (GUI) Python software that is used to implement this method is available under a GPLv3 license on ESRF GitLab, and produces a spline file that is usable with data-reduction software such as *FIT2D* or *pyFAI*.

## Abstract 2

In this article, Python software with a graphical user interface (GUI) for spatial-distortion correction, using any regular grid as the calibration pattern and producing spline files, is presented.

## 1. Introduction

Indirect detector systems bound on a conversion stage often suffer from various artefacts that distort measured data. Among them, non-uniform pixel response, readout noise, spatial deformation and blurring owing to convolution with a point spread function (PSF) may be functions of detector position. All these distortions are measurement artefacts which should be avoided or corrected, if possible before data reduction. Without correction, these artefacts reduce the data quality and may prevent accurate data processing, fitting and interpretation.

Distortions that are sufficiently constant in time can be measured using a calibration pattern which produces a predictable result. Care must be taken that the conditions where the calibration test has been performed are as close as possible to the conditions where real data are acquired. Here, we focus on spatial distortion, which can come from various sources in the optical pathway of the detector system that are more or less controlled. In particular, the result of the calibration procedure will be benchmarked with a fiber-optics-coupled CCD camera used for small-angle X-ray scattering (SAXS) acquisitions. The fiber-optic faceplate that couples the visible light from the phosphor to the CCD sensor is here the main source of spatial distortion.

This calibration procedure is important in various domains to improve the spatial resolution of images from 2D detectors. For example, an azimuthally averaged scattering pattern needs good spatial resolution to avoid smearing (Stanton *et al.*, 1992 ▸; Tate *et al.*, 2005 ▸; Mingard *et al.*, 2011 ▸), and in medical imaging accurate measurements need to be carried out on the images (Spector *et al.*, 1972 ▸; Muehllehner *et al.*, 1980 ▸; Chakraborty, 1987 ▸). In the X-ray field, *FIT2D* (Hammersley *et al.*, 1994 ▸; Hammersley, 2016 ▸) has been used for a long time for this kind of calibration and correction of 2D patterns, using regular orthogonal hole grids and splines. Several software packages rely on the distortion splines created by *FIT2D* to perform distortion correction, like *SPD* (Boesecke, 2007 ▸) or *pyFAI* (Kieffer, 2012 ▸). However, it appears that there is no software for creating this file independently, or using non-rectangular grids. So, a new specific Python software, with a graphical user interface, has been developed and made available for the community.

## 2. Calibration-grid design

The calibration pattern is typically an opaque mask with a regular 2D grid of transparent holes, which produces a known pattern on the detector. The mask is placed as close as possible to the detector and then illuminated. The difference between the ideal 2D positions and the measured positions is a measure of the distortion of the detector system.

The hole size and shape must allow an accurate and unambiguous determination of the position, independent of the alignment of the grid on the detector. In addition, the machining of the mask must be performed with the highest precision. Thus, circular holes are the easiest shape to make and fit, and still correspond to usual practice (Mingard *et al.*, 2011 ▸; Tate *et al.*, 2005 ▸). A circle has the advantage of having an infinite number of symmetry axes and a center that can be determined using various algorithms. Hammersley *et al.* (1994 ▸) show that the minimum grid-hole diameter should be >6σ of the detector PSF.

The calibration-grid shape has been discussed in the literature (Hammersley *et al.*, 1994 ▸). Non-regular grids might be of interest for oversampling the regions where the data are of most importance, or where the distortion is known to be greater. However, the use of regular grids is still common practice and is the best choice for general-purpose detectors, where the relative importance of measured data is not known *a priori*. Regular grids also have the advantage of the hole position being easier to find using an iterative algorithm. The distance between holes must allow a good separation of them along the recorded image while maintaining a large number of holes to improve the sampling over the distorted area. The Nyquist criterion suggests that the highest frequency in the measured distortion function is half the sampling frequency. Therefore, higher-frequency distortions will not be corrected using this method. Regarding these constraints, triagonal grids (holes organized on the summits of equilateral triangles) are the best because the sampling of spatial frequencies is more uniform for all directions and this arrangement maximizes the number of holes over a specific area. However, in recent decades, orthogonal grids have been the most used (Hammersley *et al.*, 1994 ▸; Spector *et al.*, 1972 ▸; Stanton *et al.*, 1992 ▸; Chakraborty, 1987 ▸; Despres *et al.*, 2007 ▸; Muehllehner *et al.*, 1980 ▸; Vijayan Asari *et al.*, 1999 ▸; Mingard *et al.*, 2011 ▸; Tate *et al.*, 2005 ▸).

## 3. Spatial-distortion measurement and correction

The spatial-distortion-function measurement process has been subdivided into the following operations: (1) determination of the origin and initial base vectors, (2) finer determination of the base vectors, (3) determination of each bright-spot position in the image, (4) pixel-size calculation and optional correction, and (5) spline fitting. Then, from the splines, the pixel-wise correction matrix can be computed and applied for distortion correction.

### 3.1. Peak-center determination

Each hole of the grid is visible as a bright spot on the detector image. From an initial guess of the position and a search radius, a sub-region of the image is considered. For practical reasons, this sub-region is square and not circular. In this sub-region, the center of the bright spot is determined by the weighted average of the bright-pixel positions. Pixels are called ‘bright’ when the intensity is above a certain threshold. 

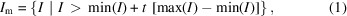

where *I* and *I*_m_ are the sub-region matrix and the bright-spot ensemble, respectively, and *t* is the threshold coefficient. A typical value for the threshold is 0.6. Then, the bright-spot center is estimated as the weighted average of these pixels: 

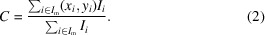

This method is very simple but works for any hole shape with at least two axes of symmetry. The reason why the condition is determined over the sub-region and not on the whole image is because the spots might have different absolute intensities, depending on how the grid is illuminated.

Sometimes, the sub-region contains no peaks, for example if the peak is hidden by a beamstop or close to the boundary. The ‘no peak’ condition is determined when all or no pixels are selected for the weighted average, or when the maximum is not statistically different from the baseline: 

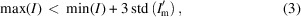

where *I*′_m_ is the complementary ensemble of *I*_m_ and std refers to standard deviation.

### 3.2. Base-vector determination

From an initial guess of the base vectors (*O*, *A*, *B*) provided by the user, the peak positions are first refined using the method described in Section 3.1. The first approximation of base vectors **e**_1_ and **e**_2_ is carried out as follows: **e**′_1_ = *A*–*O* and **e**′_2_ = *B*–*O*.

Then, to refine the base-vector approximation, all bright spots along **e**′_1_ and **e**′_2_ from the origin are detected. The fine approximation of **e**_1_ and **e**_2_ is then determined by the average vector between the bright spots: 

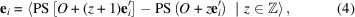

where PS(*P*) is the peak-search algorithm described in Section 3.1 and *P* is the initial guess.

### 3.3. Full distortion grid

Once a fine approximation of the base vector has been determined, the regular grid (*G*) on the image is computed by all combinations of the two base vectors: 

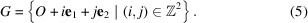

Then, all peak positions are searched for each point of the regular grid as an initial guess, and the displacement of the bright spots according to the regular grid is measured: 

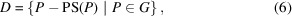

where *D* is the spatial-distortion ensemble, sampled at all regular-grid positions. However, some bright spots might be ill defined. For example, the shape may be cut by a beamstop, the intensity might be too low to distinguish the bright spot or the spot might be too close to the boundaries. The spatial-distortion ensemble is then filtered to remove these abnormalities, assuming that the distortion is smooth over the detector. The condition to assign one distortion measure as abnormal is that the displacement along one direction is farther than the average displacement of close positions, with an acceptance band of three times the standard deviation. 

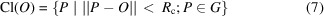

and 

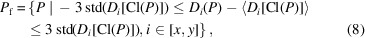

where Cl is the ensemble of points close to the considered point, *i.e.* closer than the coherence radius (*R*_c_), and *P*_f_ is the ensemble of filtered points, where the measured displacements are trusted.

### 3.4. Pixel-size calculation

**Figure 1:**
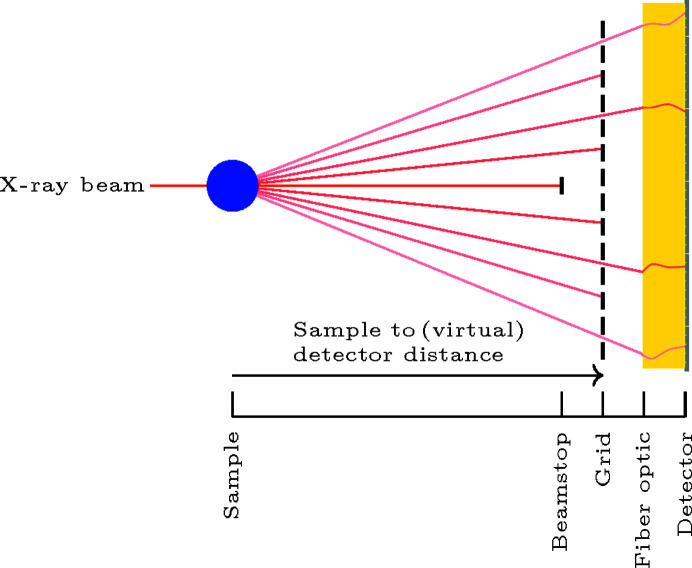
Relative positions of sample, grid, fiber optic and detector. The detector is seeing a deformed calibration grid.

**Figure 2:**
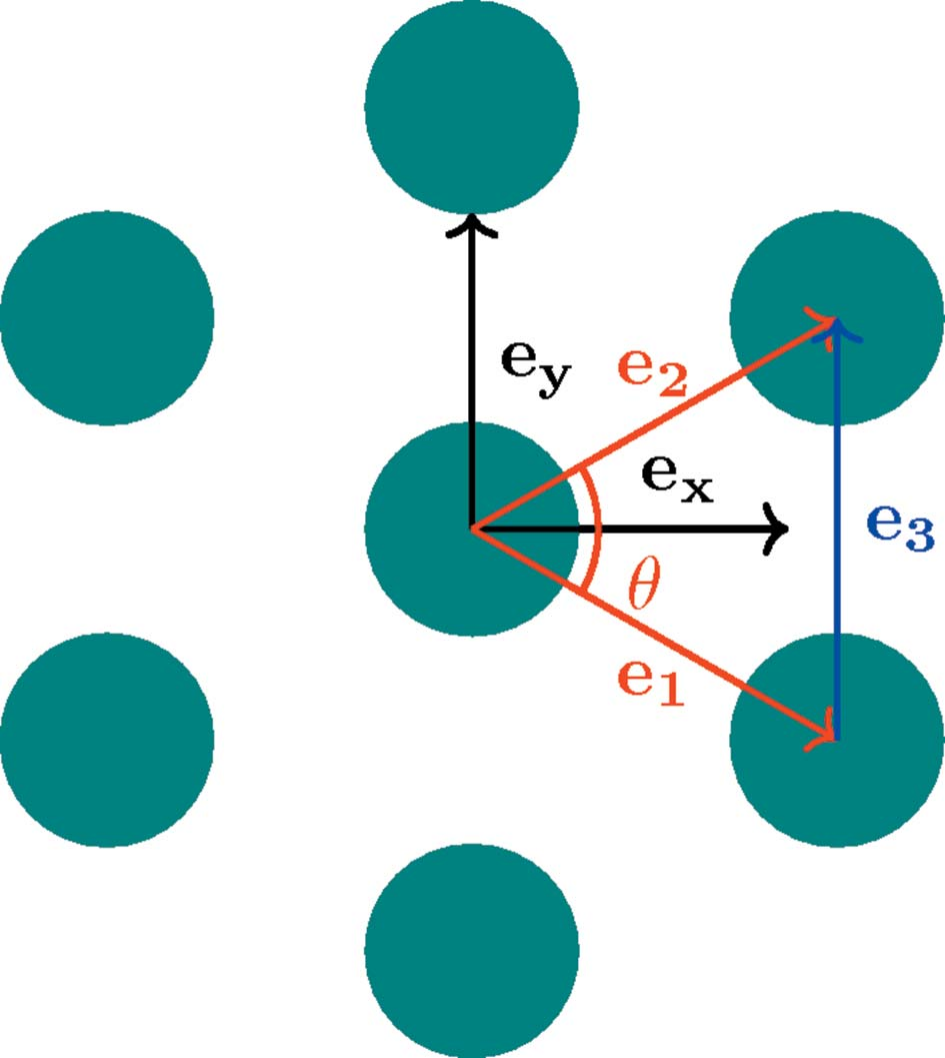
Ill-chosen base vectors for pixel-size calculation (in red) and an alternative base vector (in blue).

On some detector systems, the apparent pixel sizes are not exactly known, or can vary due to distortion in the detector optics. This is a typical problem with indirect X-ray conversion, *e.g.* coupling of an X-ray converter with optical lenses or fiber optics, which is usually the main source of spatial distortion. In these cases, the X-ray sensitive area is generally different from the image-sensor area. A possible solution is to calibrate the pixel size to an undistorted virtual detector, knowing the physical distance between bright spots on the grid. The pixel size and distances then apply to a virtual detector with an active area coinciding with the position of the calibration grid (see Fig. 1 ▸). The equation system to be solved is 

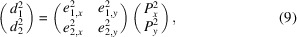

where *d*_1_ and *d*_2_ are the physical lengths of **e**_1_ and **e**_2_, respectively, and *P*_*x*_ and *P*_*y*_ are the pixel sizes along *x* and *y*. However, for certain orientations of the base vectors, this system is ill conditioned (see Fig. 2 ▸). For example, if *e*_1,*x*_ ≃ *e*_2,*x*_ and *e*_1,*y*_ ≃ −*e*_2,*y*_, because of the squared values, the two equations will be almost the same.

In this case, a second system can be used, knowing the angle between base vectors of the regular pattern (θ): 

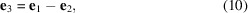




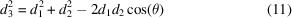

and 

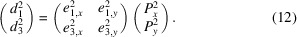

In practice, one can choose the best system to solve on the basis of the condition number of the matrices. The discrepancy between the measured and the theoretical pixel size can be corrected in the displacement matrix. This additional correction then allows one to use the theoretical pixel size for further calculations after the image has been distortion corrected. It is convenient to introduce the pixel-size correction at this step, especially for detectors with square pixels. This additional correction simplifies further calculations because it reduces the pixel size to a single parameter, which can be set to any value. Let us define it as *r* = [*P*_*x*_/*P*_*x*,th_; *P*_*y*_/*P*_*y*,th_]. The new displacement grid to be considered is 

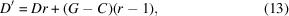

where *C* is the coordinate of the expansion/contraction center, usually the center of the image.

### 3.5. Spline calculation

Up to now, the displacement has been determined at the sample points, which are the regular-grid positions. However, in order to correct an image, the displacement along each axis has to be determined for each pixel in the image. So, the measures need to be interpolated over the whole image area. One convenient method is to use the bicubic spline function. The coefficients can be fitted and the number of knots minimized in order to obtain a smooth displacement function while keeping a good representation of the displacement samples.

However, this method will not work directly for sharp transitions between different parts of the image: for example, on detectors with multiple modules, to correct for module misalignment. To perform this kind of correction, a spline function needs to be computed for each module of the detector instead of the whole image area.

### 3.6. Image correction

Once the two spline functions have been calculated, they can be saved and reused to correct the acquisitions by the detector system. To perform the image correction, the displacement for each pixel is calculated first: 

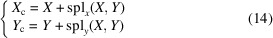

where *X* and *Y* are the coordinates matrix of all the pixels, *X*_c_ and *Y*_c_ are the displaced coordinates, and spl_*x*_ and spl_*y*_ are the displacement splines along *x* and *y*, respectively.

**Figure 3:**
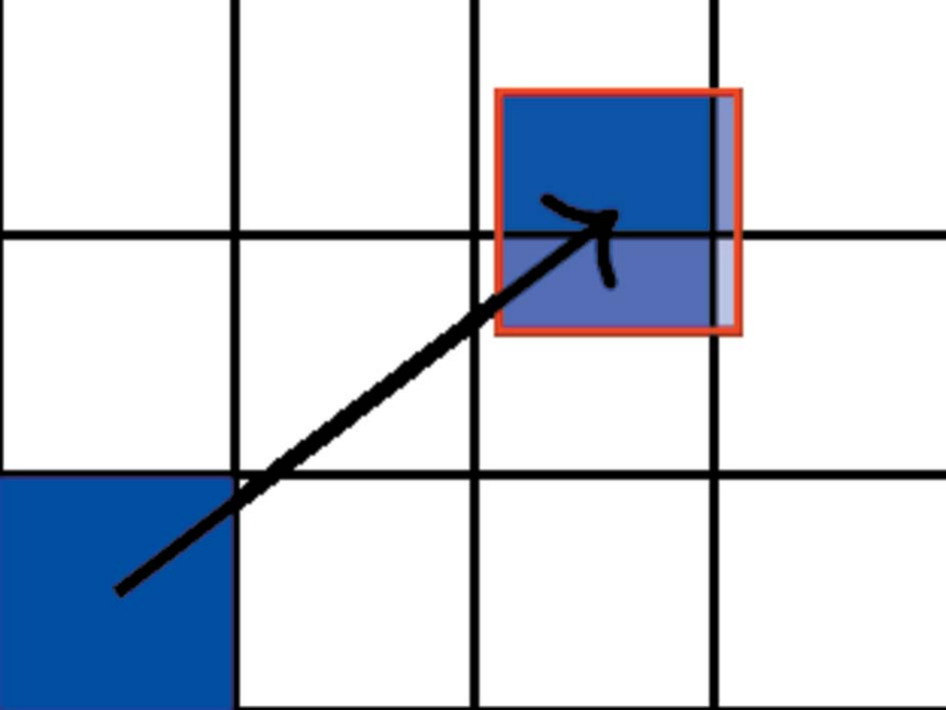
Pixel displacement and intensity redistribution according to area covered on an undistorted image.

Of course, there is no reason why all pixels should be moved by an integer number of pixels. In the general case, one pixel is spread over four pixels (see Fig. 3 ▸). The intensity from the initial pixel is weighted by the area covered by the displaced pixel on the new pixel. The weight of each new pixel is normalized so that the sum of weights on each pixel is equal to 1, *i.e.* the area of each pixel is equivalent.

In practical implementation, the images are converted to indexed vectors and the transformation is a sparse matrix. Non-null elements in this matrix at position (*i*, *j*) are the contribution weight of pixel *i* of the raw image to pixel *j* of the corrected image. Finally, to apply the correction, a matrix multiplication between the raw-image indexed vector and this sparse transformation matrix is performed. If *N* is the number of pixels in the images, the transformation-matrix size is *N* × *N* and the matrix has at most 4*N* non-null elements.

## 4. Discussion

Spatial distortion is only one among many corrections that need to be carried out on 2D detectors. In particular, any intensity correction needs to be performed before spatial-distortion correction, because this correction rebins the image (see Section 3.6) and the effective pixel area is changed. Corrections such as (but not limited to) dark-image subtraction, flat-field correction and hot-pixel masking also have to be performed before spatial-distortion correction.

The method used to interpolate the distortion map over all the pixels on the detector using splines has the advantage of smoothing the sampled map, and then being less sensitive to the exact determination of the bright-spot center. However, this prevents sharp transition in the distortion function. For sharp transitions such as misalignment of modules in a modular detector, the spatial distortion has to be measured on the whole detector but the spline functions need to be determined for each module, allowing discontinuities between them.

## 5. Practical software implementation

The ideas presented above have been implemented in a Python software named *SpatDistCalib* (Chèvremont, 2022 ▸), with the help of *NumPy* (Oliphant, 2021 ▸), *SciPy* (Virtanen *et al.*, 2020 ▸) and *Matplotlib* (Hunter & Droettboom, 2021 ▸) libraries. The *FabIO* (Knudsen *et al.*, 2013 ▸) library is also used to read the raw image from a file. This library can read various file formats from a large range of detectors. Once read, the image is transformed into a *NumPy* array, allowing one to use the functions of this software with images from almost any source.

The user is requested to provide the origin and the base vectors by clicking on the origin (*O*), *O* + **e**_1_ and *O* + **e**_2_. The user input is refined by the method described in Section 3.2. The software then displays the regular grid, as well as the actual grid detected on the image.

The user is then requested to provide the physical length of the base vectors, *d*_1_ and *d*_2_, as well as the angle between them, in order to deduce the pixel size in both directions. If pixel-size correction is enabled, the software will introduce the enlargement or contraction to correct the pixel size to the ideal pixel size. After that, the software displays the displacement maps, the splines computed and the errors, as well as the calibration image corrected by the splines, with the regular grid superimposed. The splines can be saved in text format, which is usable by online data-reduction software, such as *SPD* (Boesecke, 2007 ▸), *FIT2D* (Hammersley, 2016 ▸) or *PyFAI* (Kieffer & Karkoulis, 2013 ▸; Kieffer & Wright, 2013 ▸).

## 6. Result of calibration correction

A full example of distortion correction applied on a SAXS pattern taken using a FReLoN detector at the ID02 beamline at ESRF (Narayanan *et al.*, 2018 ▸; Labiche *et al.*, 2007 ▸) is available in Appendix *A* [App appa].

**Figure 4:**
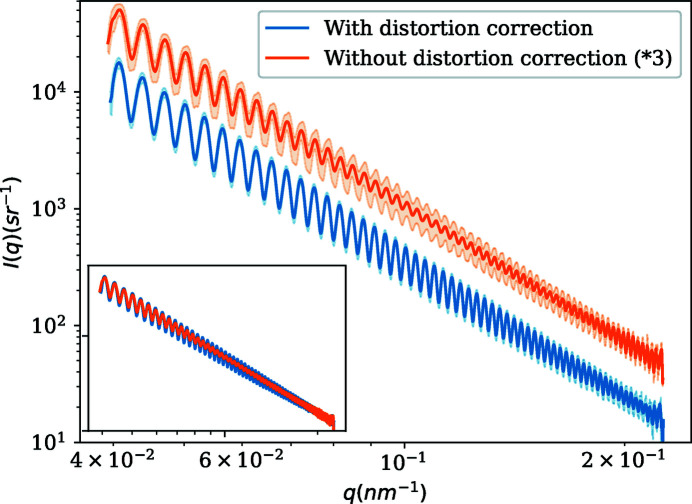
One-dimensional SAXS curves before and after distortion correction. Shaded areas represent the standard deviation estimated from azimuthal regrouping. For display purposes, the curve without distortion correction has been shifted upward. The inset shows the curves without standard deviation or shift.

Fig. 4 ▸ shows the 1D curve determined from azimuthal regrouping before and after applying the distortion correction for a sample of uniform spherical particles (polystyrene latex). The shaded areas represent the standard deviation of the average from azimuthal regrouping. For display purposes, the curve without distortion correction has been shifted upwards.

A comparison between the curves shows that the spatial-distortion correction enhances the data quality obtained by reducing the smearing as well as the standard deviation. The oscillations are better resolved and the relative amplitude seems constant over a wide dynamic range. For the lowest scattering vector magnitude *q*, the number of pixels is small and the pixels are closer, so they should be less sensitive to spatial distortion. Even here, the standard deviation is clearly improved. As the curve goes higher in *q*, the smearing effect of spatial distortion is more and more pronounced, and the relative standard deviation increases. On the other hand, the relative standard deviation seems to stay more constant after the spatial-distortion correction.

## 7. Conclusions

In this article, a method to measure image deformation and calibrate 2D detectors using a calibration grid has been described in detail. The proposed method is able to correct low-frequency deformations of an image, measure effective pixel size and correct the size to the theoretical one, so that the corrected image is like that taken by a virtual perfect sensor at the calibration-grid position. The only hypothesis on the calibration grid is that it is a regular grid, described by two base vectors.

This method has been implemented and provided to the user community as a Python software package. The method has been tested on a 2D detector that exhibits image deformation, FReLoN 4M at ID02 beamline, ESRF, demonstrating how the calibration grid can be reshaped as a regular grid using spatial-distortion correction splines. The correction has also been used on monodisperse polystyrene beads, which show a large number of oscillations. Applying this correction clearly improves the resolution of these oscillations and reduces the standard deviation of the averaged 1D curve.

To conclude, this software provides a standalone alternative to generate displacement files for the spatial calibration of 2D detectors. This software also allows one to use any kind of regular grid for the calibration, not just orthogonal ones. The Python source code is available for the community and can be easily extended or improved for specific needs. The spline files generated by this software can be used directly with software like *FIT2D*, *SPD* or *pyFAI* for distortion correction. The calibration has been demonstrated to work well using triagonal calibration grids, and the spatial-distortion correction, when applied on samples exhibiting oscillations over a wide dynamic range, has been shown to recover them accurately on the 1D azimuthal reduced curve.

## Figures and Tables

**Figure 5:**
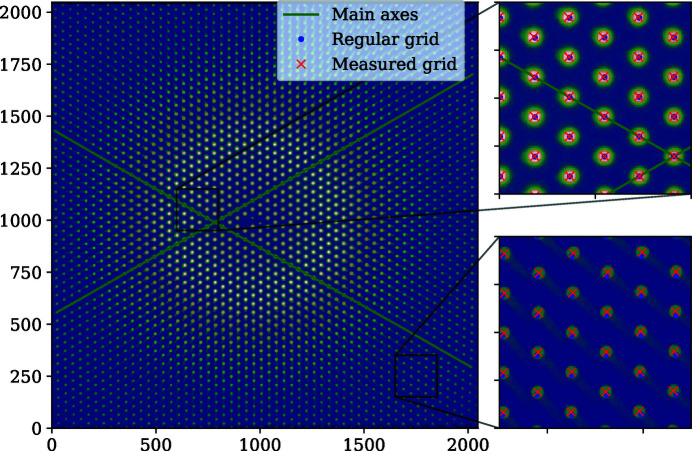
An image of the calibration grid. The colors represent pixel intensity on a log scale. The green axes are the main axes along which **e**_1_ and **e**_2_ are estimated. Regular grid refers to the grid generated using the base vectors. Measured grid is the bright-spot positions determined using the peak-search method. The grid is illuminated by monochromatic 12 230 eV X-rays, broadened using diffusion of a dilute colloidal suspension, 10 m away from the detector.

**Figure 6:**
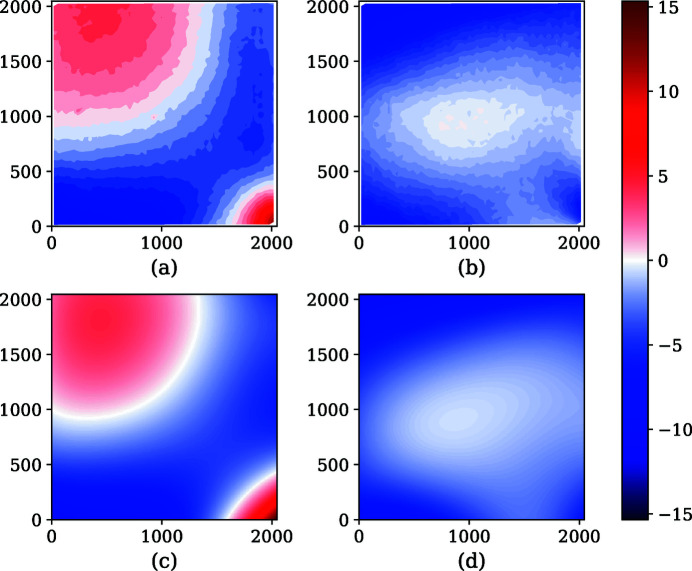
(*a*) Measured displacement along *x*. (*b*) Measured displacement along *y*. (*c*) The spline function for displacement along *x*. (*d*) The spline function for displacement along *y*. All units are in pixels.

**Figure 7:**
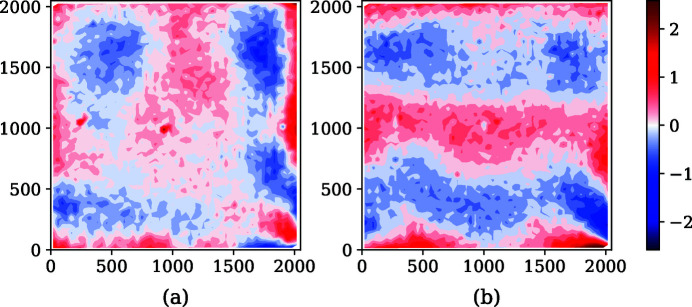
(*a*) Errors between the spline function and displacement measured along *x*. (*b*) Errors between the spline function and displacement measured along *y*.

**Figure 8:**
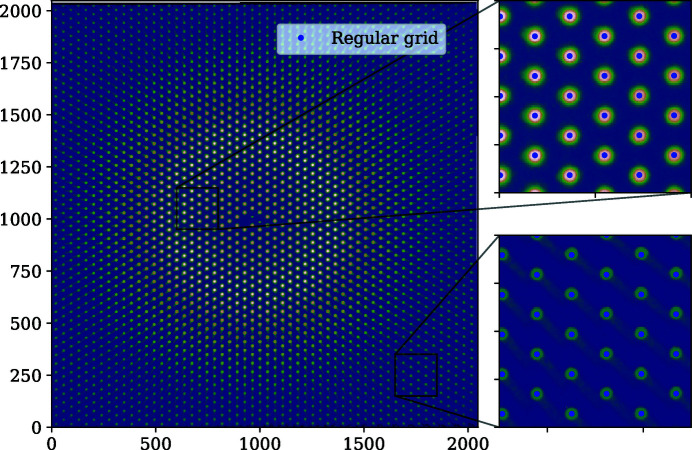
The calibration-grid image corrected by the splines, with the regular grid superimposed.

**Figure 9:**
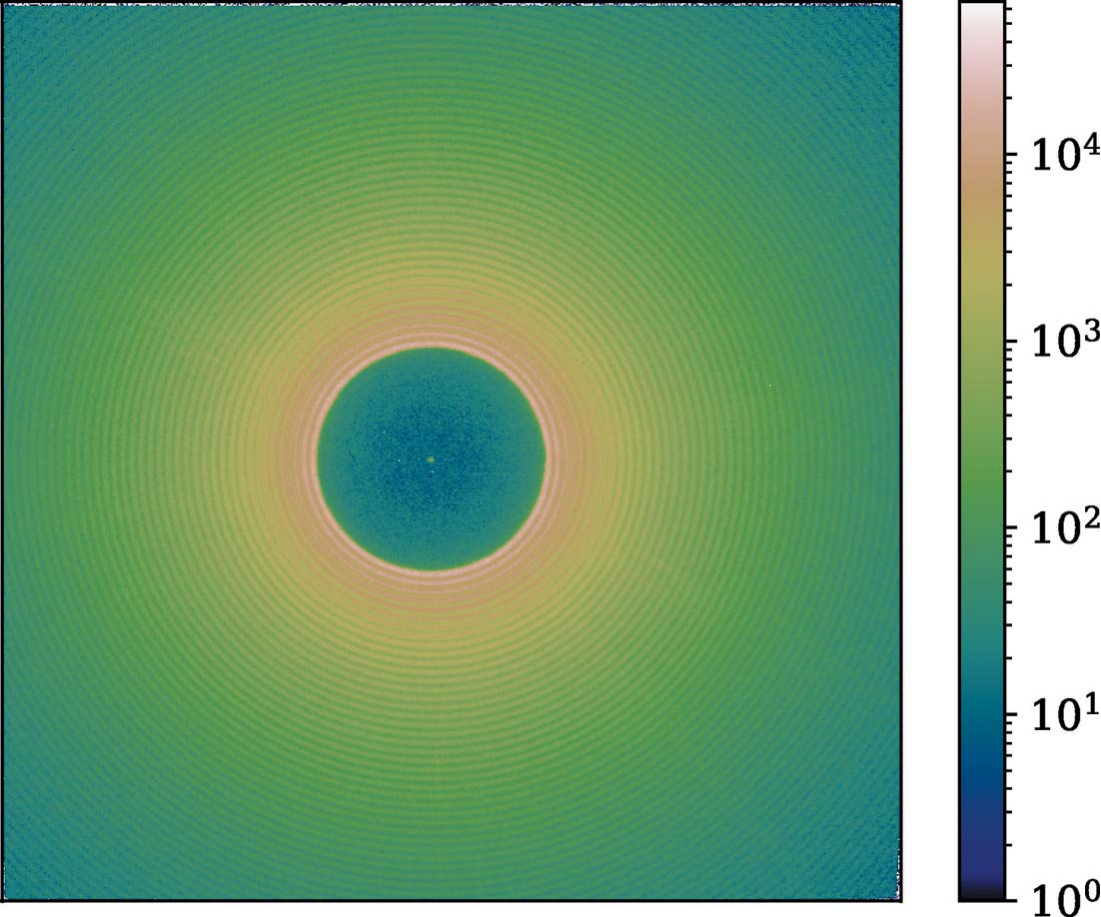
A SAXS pattern of 2 µm polystyrene beads, taken at a sample-to-detector distance of 10 m, with an exposure time of 3 s (after dark-image subtraction and flat-field correction). A large beamstop (12 mm) is used for measuring the intensities at larger scattering angles with high accuracy.

**Figure 10:**
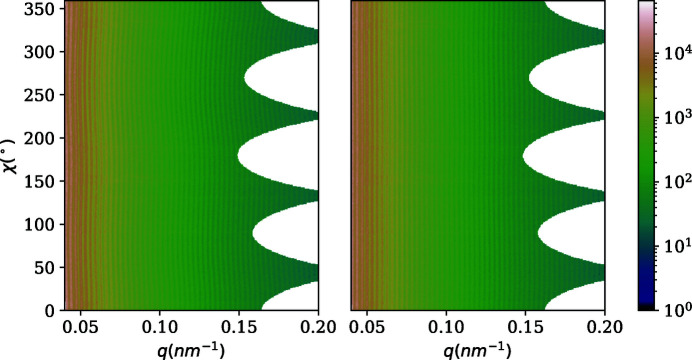
An azimuthally regrouped SAXS pattern of polystyrene beads, before (left) and after (right) distortion correction.

## Appendix A Results of calibration correction

The concepts and software implementation presented above have been applied to the distortion correction of isotropic small-angle scattering data taken with a custom FReLoN camera, used on the ID02 beamline of ESRF (Narayanan *et al.*, 2018 ▸; Labiche *et al.*, 2007 ▸). This camera has 24 µm square pixels, which are almost 1:1 coupled with a fiber-optics faceplate. This faceplate is the main source of image distortion. The calibration grid is a 0.350 mm thick nickel plate with 0.290 ± 0.010 mm diameter holes, which are organized in a regular triagonal pattern. The deviation of each hole from an ideal circle is typically 0.010 mm, never exceeding 0.030 mm. The distance between edges is 0.710 ± 0.010 mm. So, the distance between hole centers is 1 mm. The grid parameters have been determined using optical microscopy and ∼2750 holes are visible on the image.

### A1. Displacement-function determination

Fig. 5 ▸ shows an image of the grid illuminated by X-rays at 12 230 eV, broadened by the diffusion pattern of a dilute colloidal suspension at 10 m from the detector. At the center of the image, the beamstop covers some holes, which leads to missing and cut bright spots. In the two zoomed areas, the regular-grid position as well as the measured position of each peak is displayed. The bright spots are well separated from each other. For the bright spots with lower intensity (further away from the center), a tail is visible. However, the intensity of this tail is below the threshold used to determine the bright-spot position and therefore does not disturb the measurement.

Several different origins have been tested. Unless the main axes contain a lot of ill-determined bright spots (cut by the beamstop, detector defects, very large displacement,…), all displacement maps determined have very similar shapes, and the values only differ by a constant, since the origin (*O*) fixes the origin of the grid with no displacement. The pixel size determined with different origins is not sensitive to the origin taken.

Fig. 6 ▸ shows the measured displacement along *x* and *y* [(*a*) and (*b*)] and the computed splines for these displacement interpolations [(*c*) and (*d*)]. On the *x* displacement map, a corner has a large displacement, caused by cutting of the fiber-optics plate. The displacements along *x* and *y* seem completely decoupled.

The spline function reflects the general shape and amplitude of the displacement map. However, splines are known to exhibit larger discrepancy close to the domain border. This is why the shape of the large deformation that is bottom right on the *x* displacement map is not very well described.

The pixel size that is computed using the physical parameters of the grid is 23.83 × 24.17 µm (*H* × *V*). As explained in Section 3.4, this non-squareness correction can be added to the displacement map, or taken into account when calculating the scattering vector magnitude (*q*) of each pixel. The first option is usually more convenient, as this simplifies the calculation of *q* and reduces the pixel size to a single value, which can be set to the theoretical one.

Fig. 7 ▸ shows the error (*i.e.* the difference) between the measured displacement and the spline that interpolates the measurements. For both functions, the error stays below one pixel for most of the central area. For the *x* displacement error, one value is higher in the middle, but this value is due to a bright spot that is partially hidden by the beamstop but not removed by the algorithm. The largest values occur at the domain boundary but stay reasonable. The maximum error is 2.4 pixels for the bottom-right *y* displacement.

Fig. 8 ▸ shows the initial calibration grid corrected using the spline functions that have been determined. The regular grid is superimposed on the image without any further adjustment. The position of all the bright spots now matchs the regular grid, even in the place where the error was large (Fig. 8 ▸, bottom right).

### A2. Application to a scattering pattern

Once the detector spatial distortion has been described, the correction can be applied to images of real data. Here, an isotropic SAXS pattern of 2 µm polystyrene beads is used to demonstrate the quality of correction (see Fig. 9 ▸, which is dark-image subtracted and flat-field corrected). The scattering pattern shows oscillations over a wide dynamic range. In order to resolve as many oscillations as possible, a large beamstop was used to avoid saturating the CCD detector close to the primary beam, and a long exposure time of 3 s was applied. A large number of oscillations can be seen by eye, from very close to the beamstop to the corner of the image. The incident-beam position is almost at the center of the image and is visible through the beamstop.

Fig. 10 ▸ shows the azimuthal regrouping of Fig. 9 ▸, before spatial-distortion correction (left) and after correction (right). The distorted raw and corrected patterns are both azimuthally regrouped, averaged and compared. Even though the center has been carefully determined before and after correction, the azimuthal regrouping still exhibits waves before correction. These waves prevent averaging of the regrouped data over all angles in order to get a high-resolution 1D curve. On the right side, the azimuthal regrouping exhibits much straighter vertical lines. As the beam center might be shifted by applying the distortion correction, the beam position after spatial-distortion correction has to be determined again.
